# 144 Generations Uniting through Movement - Good practices on intergenerational physical activity programs

**DOI:** 10.1093/eurpub/ckae114.015

**Published:** 2024-09-26

**Authors:** Berry Van Holland, Hilal Erkoca, Remo Mombarg

**Affiliations:** Hanze University of Applied Sciences, Groningen, The Netherlands; ISCA, Copenhagen, Denmark; Hanze University of Applied Sciences, Groningen, The Netherlands

## Abstract

The GUM project aimed to use traditional games, movement and sports as one way of promoting intergenerational relations and increased HEPA by setting up an innovative and sustainable GUM program including the creation of a European platform and network on EU Generations Uniting through Movement, a training tool for practitioners and trainers and a GUM app for both youth and elderly.

The concept for the GUM program was to identify, recruit, train and support a cohort of experienced practitioners who will facilitate local-scale collaboration projects between youth and elderly to increase their participation in health-enhancing physical activity and sport. For this purpose the project partners identifyied working principles for those activities (report, factsheet).

These principles served as basis for the development of an online training tool allowing practitioners all over Europe to follow the 7 modules in their own time. The training tool works on multiple aspects: 1) illustrate goals, principles and components of an intergenerational movement and physical activity program, 2) design and deliver a successful GUM program, 3) apply and develop digital literacy as support to GUM offer (using GUM app).

The miMove GUM app allows users to keep track of their activities and communicate with other users and GUM program facilitators. The GUM app has been designed to be used by both elderly and youth allowing them to engage through fun activities. It was also designed to co-create activities by facilitation of GUM program practitioners/trainers. It consists of step-by-step intergenerational movement activity examples selected by the GUM project partners.

GUM project partners have already established a European GUM network, but are also reaching out to partners across Europe and the rest of the world to join the network and exchange knowledge and experience regarding intergenerational HEPA programs.

Several products have been finalized and implemented in practice. Among them are a research report on guiding principles for intergenerational programs, a factsheet for practitioners, a training tool for practitioners and an app for practitioners and end-users. Furthermore, an online platform for the European GUM network has been realized. All these products will help to organize intergenerational HEPA programs.

